# A new extremophile ostracod crustacean from the Movile Cave sulfidic chemoautotrophic ecosystem in Romania

**DOI:** 10.1038/s41598-023-32573-w

**Published:** 2023-04-14

**Authors:** Sanda Iepure, Anna Wysocka, Serban M. Sarbu, Michalina Kijowska, Tadeusz Namiotko

**Affiliations:** 1 Emil Racoviţă” Institute of Speleology, Clinicilor 5, 400006 Cluj Napoca, Romania; 2 Faculty of Biology, Department of Evolutionary Genetics and Biosystematics, University of Gdańsk, Wita Stwosza 59, 80-308 Gdańsk, Poland; 3 Department of Biological Sciences, California State University, Chico, CA 95929 USA; 4 Department of Taxonomy and Ecology, University Babes Bolyai, Cluj Napoca, Romania

**Keywords:** Evolutionary developmental biology, Evolutionary genetics, Origin of life, Speciation, Taxonomy, Evolution, Zoology, Ecology

## Abstract

Sulfidic cave ecosystems are remarkable evolutionary hotspots that have witnessed adaptive radiation of their fauna represented by extremophile species having particular traits. Ostracods, a very old group of crustaceans, exhibit specific morphological and ecophysiological features that enable them to thrive in groundwater sulfidic environments. Herein, we report a peculiar new ostracod species *Pseudocandona* *movilaensis* sp. nov. thriving in the chemoautotrophic sulfidic groundwater ecosystem of Movile Cave (Romania). The new species displays a set of homoplastic features specific for unrelated stygobitic species, e.g., triangular carapace in lateral view with reduced postero-dorsal part and simplification of limb chaetotaxy (i.e., loss of some claws and reduction of secondary male sex characteristics), driven by a convergent or parallel evolution during or after colonization of the groundwater realm. *P.* *movilaensis* sp. nov. thrives exclusively in sulfidic meso-thermal waters (21 °C) with high concentrations of sulphides, methane, and ammonium. Based on the geometric morphometrics-based study of the carapace shape and molecular phylogenetic analyses based on the COI marker (mtDNA), we discuss the phylogenetic relationship and evolutionary implication for the new species to thrive in groundwater sulfidic groundwater environments.

## Introduction

Sulfidic ecosystems from thermal vents in deep sea, continental karst springs, and caves, are inhabited by some of the most extraordinary extremophile organisms on the planet^1–3^. In these ecosystems, the absence of light precludes photosynthesis—the chemical process by which green plants draw energy from sunlight to build carbohydrates from water and carbon dioxide. Here, chemoautotrophic microbes are at the bottom of the food chain and use a different strategy to extract energy by oxidizing hydrogen sulphide as a replacement for radiant energy from sunlight^4–6^. They not only survive in this challenging environment but also provide food for organisms of higher trophic levels.

In the last decade, deep sea thermal vents have attracted much attention due to their high diversity of chemoautotrophic prokaryotes and marine invertebrates that have adapted to wide thermal gradients, high pressure, and chemically extreme environments usually by living in symbiosis with chemosynthetic microbes, enabling them to cope with toxic waters rich in hydrogen sulphide and methane^7–9^. In contrast, in continental sulfidic cave ecosystems (SCE), which share their highly unusual nature with sulfuric deep-sea hydrothermal vents, fundamental studies on invertebrate biota are still scarce^5,10–15^. An exception is the first discovered cave ecosystem of this type, the Movile Cave in Romania, the microorganism communities of which have been exhaustively studied since its discovery^5,6,16,17^.

Sulfidic cave ecosystems caves are generally considered extreme and are characterized by warm waters, high levels of sulphide, methane, and ammonium; heavy metals (iron, zinc, and copper); and low oxygen concentrations up to hypoxia^5,18–20^. Hydrogen sulphide concentration is significantly high in SCE and was abundant in the ancient, anoxic oceans of the Proterozoic serving as an energy source for early forms of life^21^. Organisms from various phyla have colonized such toxic environments, giving rise to unique ecological communities and complex trophic networks, which are supported entirely by chemoautotrophic organisms and particularly by sulphate-reducing bacteria^7,8^. Although sulphide is generally highly toxic to most organisms, SCE host extremophile life forms that show a combination of morpho–ecological traits and metabolic and physiologic adaptations, enabling them to cope with such extreme conditions. In this regard, SCE and their fauna are considered remarkable ‘evolutionary hotspots’, which can be a model for extra-terrestrial life on Mars^22,23^.

The few available studies on sulfidic cave ecosystems aquatic invertebrates indicate that crustaceans show high diversity in several groups that hypothetically retain some archetypal features as adaptive features to cope with hypoxia and high concentrations of sulphide and methane in a similar way as their marine relatives ^5,10,11,14,15,24,25^. Moreover, they show high capacity for long-term anaerobiosis^26^ and well-developed mechanisms for sulphide detoxification^27,28^. Moreover, they show high capacity for long-term anaerobiosis^26^ and well-developed mechanisms for sulphide detoxification^27,28^. These subterranean species, even representing phylogenetically distant phyla, have attained striking similarity and show typical ‘regressive’ characters, e.g., reduction or absence of eyes, loss of pigment, elongated body shape and appendages, modifications of sensory organs, and slow metabolism^29^.

Among crustaceans, ostracods are only mentioned as being present in SCE as a group but neither data on species diversity nor taxonomic descriptions of new species or species exclusively associated with hydrogen sulphide-rich continental groundwaters are currently available^5,11,14^. Ostracoda are an ancient class dating back to the Early Ordovician or Late Cambrian period (~ 505 to 485 Mya)^30^. They are small crustaceans with the body enclosed in a calcified bivalve shell that completely covers the entire animal. They generally feed on aquatic bacteria, fungi, algae, and detritus.

Studies on ostracod evolution and adaptive radiation have a long history. In particular, the functional morphology of easily fossilizing calcitic carapaces, and their adaptive response to environmental conditions, have been studied intensively from empirical and theoretical viewpoints^31^ (references cited therein). The shape, ornamentation, and size of the ostracod carapace have often been subjected to evolution in the same direction in distinct and unrelated species but sharing similar environmental pressures^32^. Such similarities between organisms for reasons other than inheritance from a common ancestor is termed homoplasy and is caused by either convergent or parallel evolution^33^. Convergence may lead to homeomorphy, which is defined as similarity affecting the whole outer appearance to such a degree that one organism may be mistaken for the other.

Homoplasy (or its special case homeomorphy) is an important issue in ostracod evolutionary biology as homoplastic similarities, particularly occurring in reasonably close phylogenetic groups, can make phylogenetic analysis more challenging^31–34^. Morphological homoplasy is assumed to act with preference on those structures that have the highest probability to become advantageous for a species living in a certain environment.

Here, we describe a new cave ostracod species thriving in sulfidic-rich waters of the Movile Cave in southeast Romania. We use geometric morphometrics related to the carapace shape of the new species in comparison with its closest relatives of the genera *Pseudocandona* Kaufmann and *Typhlocypris* Vejdovský and DNA sequences of the mitochondrial cytochrome c oxidase subunit I (*COI*) gene to infer the phylogenetic relationships of the new species.

## Results

### Taxonomic account

Family: Candonidae Kaufmann, 1900

Subfamily: Candoninae Kaufmann, 1900

Genus: *Pseudocandona* Kaufmann, 1900

*Pseudocandona* *movilaensis* [Iepure, Wysocka and Namiotko] sp. nov.

***Type*** ***material.*** Holotype (NSMT-Pol H-837) female and one paratype female (ICHUM-6177) are deposited in the National Museum of Nature and Science, Tsukuba (NSMT). Allotype male is dissected in glycerin with limbs mounted on a permanent slide, and left valve stored dry in a micro-paleontological cell. A paratype female (ISER—F20) deposited at the Institute of Speleology “Emil Racoviţă” (Cluj Napoca, Romania) dissected in glycerin and limbs mounted on a permanent slide; left valve stored dry in a micro-paleontological cell. Other paratypes, ca. 40 specimens in tubes with alcohol (ISER F21—61), one male (ISER—M62) and one female (ISER—F63) dissected on permanent glass slides with valves kept dry in micropaleontological slides as well as empty valves mounted on SEM stubs) are housed in ostracod collection in the Institute of Speleology “Emil Racoviţă” (Cluj Napoca, Romania) (SI) and Department of Evolutionary Genetics and Biosystematics of the University of Gdansk (TN). Three paratype specimens were used for DNA analysis. Sequences originated from these paratypes are deposited at GeneBank (see Table S1 for accession numbers).

LSID Zoobank: https://zoobank.org/NomenclaturalActs/8bb34881-00e3-46a4-8297-9e6691fc9b4e.

***Type*** ***locality*** ***and*** ***habitat.*** Sulfidic lake in Lake Room, Movile Cave (Romania) (43°49′36.38′′ N, 28°33′43.48′′ E, 24 m a.s.l.).

***Etymology.*** The name of a new species is an adjective derived from the name of the type locality, Movile Cave near Mangalia, Constanţa County, south-east Romania.

**Figure 1:**
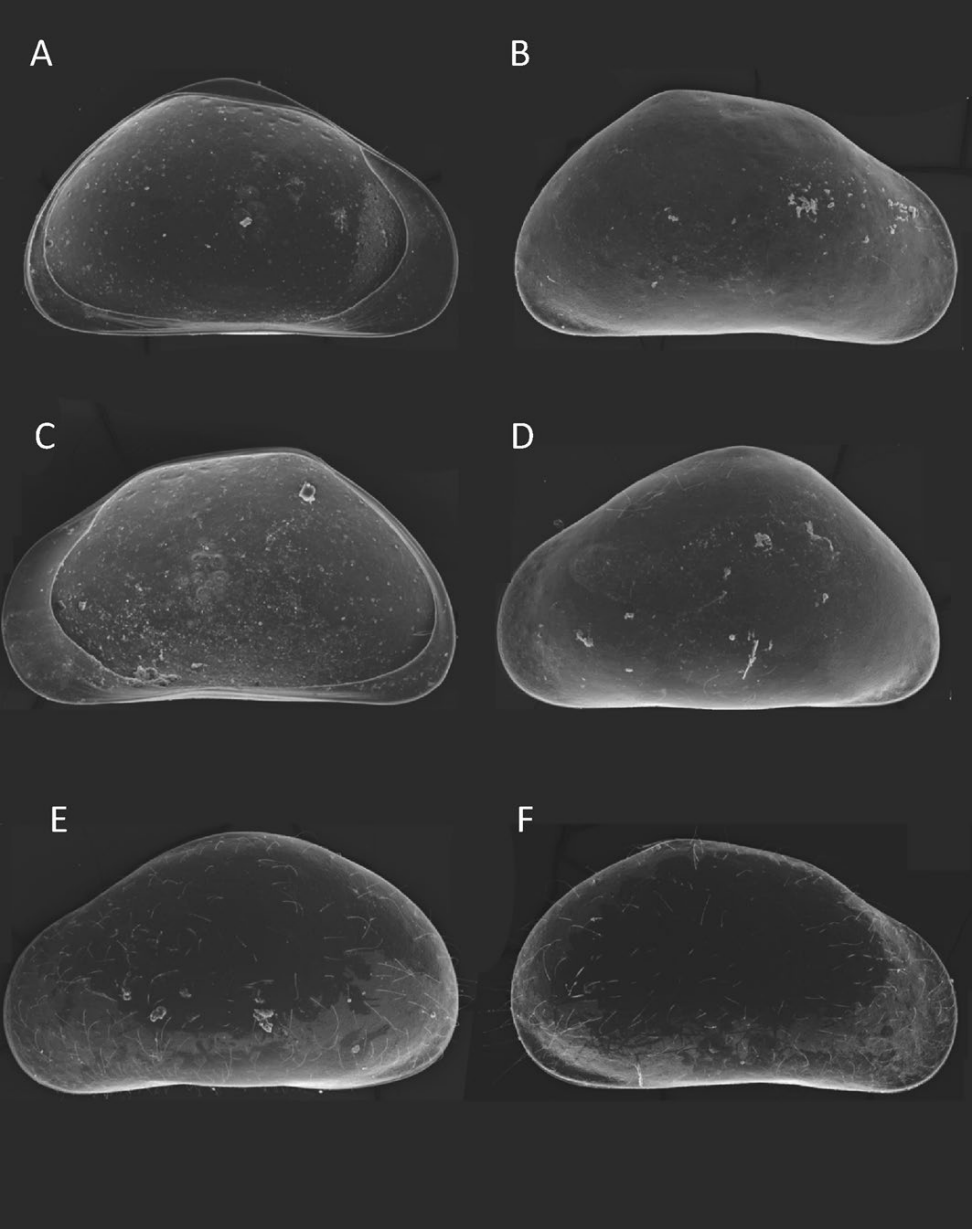
*Pseudocandona* *movilaensis* sp. nov. (**A**) Internal view of female left valve. (**B**) External view of female right valve. (**C**) Internal view of female right valve. (**D**) external view of female left valve. (**E**) External view of male left valve. (**F**) External view of male right valve (scale—200 µm).

**Figure 2:**
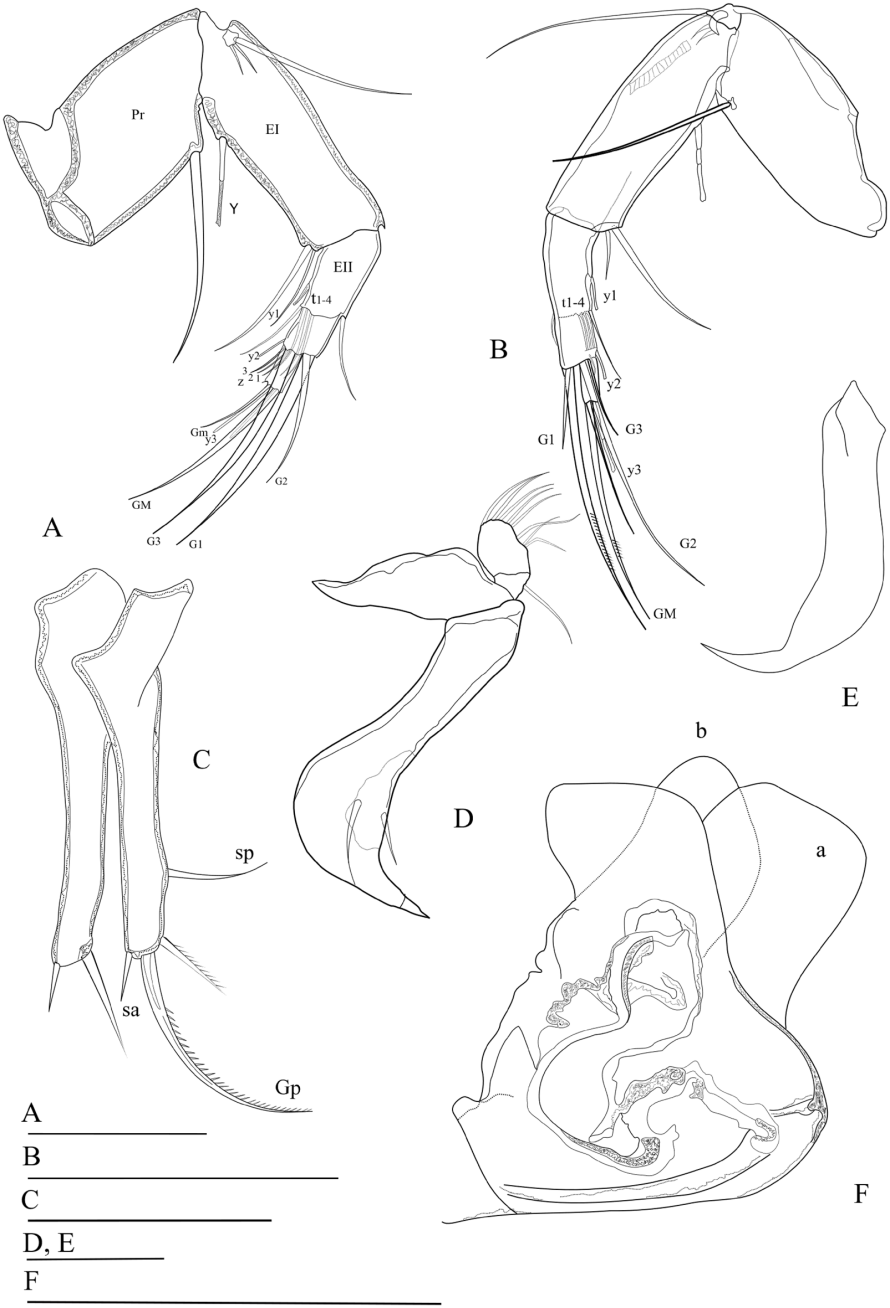
*Pseudocandona* *movilaensis* sp. nov. (**A**) Female second antenna. (**B**) Male second antenna. (**C**) Female uropodal rami. (**D**) Male right clasping organ (fifth limb palp). (**E**) Male left clasping organ (fifth limb palp). (**F**) Hemipenis. Scale bars (100 µm).

***Diagnosis.*** *Pseudocandona* *movilaensis* sp. nov. is distinctive from its congeners by the following set of morphological characters: (1) Carapace and left valves of both males and females as well as juveniles with high dorsal arch, giving a triangular shape in lateral view (Fig. 1, Figs. S1, S2). (2) Female antennal claws G_1_ = 1.6× and G_M_ = 1.3× the length of penultimate endopodial segment (Fig. 2A). (3) Male antenna with divided 2nd endopodial segment but with no male bristles (Fig. 2B, Fig. S4). (4) Male antennal claws G_2_ = 1.6×, G_M_ = 1.3× and z_1_ = 0.94× the length of (undivided) penultimate segment (Fig. 2B, Fig. S4). (5) Mandibular palp 2nd segment with 3 (setal group) + 1 + beta setae on the inner edge, and penultimate segment with gamma seta smooth (not plumose) (Figs. S3, S4). (6) Male fifth limb endopodite palps (clasping organs) asymmetrical (Fig. 2D,E, Fig. S4). (7) Seventh limb (cleaning leg) four-segmented, protopodite with three setae, penultimate segment lacking f seta and terminal segment with two long setae h_2_ and h_3_, and one short and slightly curved h1 seta (Figs. S3, S4). (8) Uropodal ramus of both sexes with posterior claw G_p_ remarkably reduced, less than half of anterior claw G_a_ (Fig. 2C, Figs. S4, S5). (9) Inner lobe (b) of hemipenis distally broadly rounded, with a distinct, acuminated expansion oriented to the postero-ventral end of the body (Fig. 2F, Fig. S5).

***Description*** (for the full description see Supplementary material).

### Geometric morphometrics of the valve shape

**Figure 3:**
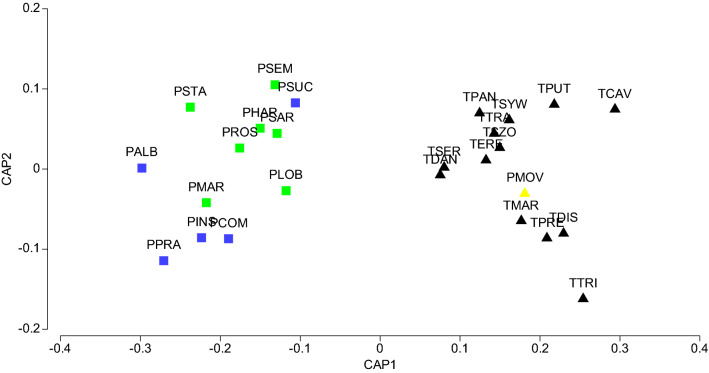
Canonical analysis of principal coordinates (CAP) plot of geometric morphometrics data of the valve shape, showing the position of *Pseudocandona* *movilaensis* sp. nov. (yellow triangle) based on its morphometric resemblances with the species of the genus *Typhlocypris* (black triangles) and those of the two species-groups of the genus *Pseudocandona* (green squares = *compressa* species-group, blue squares = *rostrata* species-group). For the species codes see Table 1.

To predict the genus/species-group to which our *Pseudocandona* *movilaensis* sp. nov. may be classified based exclusively on the valve shape, we used Canonical Analysis of Principal Coordinates (CAP) on the distance matrix from the B-spline geometric morphometrics analysis (Fig. 3). The results of CAP of the discrimination between three groups of species which were selected as morphologically the closest based on the limb morphology (*Typhlocypris* vs. *Pseudocandona* ex gr. *compressa* vs. *Pseudocandona* ex gr. *rostrata*, see Table 1) show that the first squared canonical correlation was relatively large (0.904) and indeed the first canonical axis clearly separated the stygobitic species of the genus *Typhlocypris*, all having triangular valve shape in lateral view. This was the most distinct group, which had 100% allocation success under cross-validation. The other two groups of the genus *Pseudocandona* (gr. *compressa* and gr. *rostrata*) were hardly distinct from one another (Fig. 3), although their allocation success rates were still considerably large (at 80.0% and 85.7%, respectively). The second canonical axis had a much smaller eigenvalue (0.127) and there is actually no separation of the three groups along the second axis.

**Table 1:** Species used in genetic and geometric morphometry analyses with information on the origin of data used (with valve outline source references and sample localities).

Species	Code	Geometric morphometrics Valve outline source reference	Genetic studies Origin of the studied specimens
*Pseudocandona* *movilaensis* sp. nov	PMOV	Present paper	Movile Cave, Romania
Genus *Pseudocandona* Kaufmann, 1900 species-group *compressa*			
*P.* *albicans* (Brady, 1864)	PALB	Meisch (2000)	Gdansk, Poland and Oslo, Norway
*P.* *compresa* (Koch, 1838)	PCOM	Meisch (2000)	Lake Ptasi Raj-Gdansk, Poland
*P.* *insculpta* (G.W. Müller, 1900)	PINS	Meisch (2000)	
*P.* *pratensis* (Hartwig, 1901)	PPRA	Meisch (2000)	
*P.* *sucki* (Hartwig, 1901)	PSUC	Meisch (2000)	
Genus *Pseudocandona* Kaufmann, 1900 species-group *rostrata*			
*P.* *hartwigi* (G.W. Müller, 1900)	PHAR	Meisch (2000)	Lake Otomińskie-Gdansk, Poland
*P.* *lobipes* (Hartwig, 1900)	PLOB	Meisch (2000)	
*P.* *marchica* (Hartwig, 1899)	PMAR	Meisch (2000)	Oslo, Norway
*P.* *rostrata* (Brady & Norman, 1889)	PROS	Meisch (2000)	
*P.* *sarsi* (Hartwig, 1899)	PSAR	Meisch (2000)	
*P.* *semicognita* (Schäfer, 1934)	PSEM	Meisch (2000)	
*P.* *stagnalis* (Sars, 1890)	PSTAS	Meisch (2000)	
Genus *Typhlocypris* Vejdovský, 1882			
*T.* *cavicola* (Klie, 1935)	TCAV	Klie (1935)	
*T.* *danubialis* (Iepure et al., 2007)	TDAN	Iepure et al. (2007)	
*T.* *dispar* (Hartmann, 1964)	TDIS	Hartmann (1964)	
*T.* *eremita* (Vejdovský, 1882)	TERE	Iepure et al. (2007)	Gilau, Romania
*T.* *marmonieri* (Namiotko & Danielopol, 2004)	TMAR	Namiotko & Danielopol (2004)	
*T.* *pannonicola* (Löffler, 1960)	TPAN	Löffler (1960)	
*T.* *pretneri* (Danielopol, 1978)	TPRE	Danielopol (1978)	
*T.* *puteana* (Klie, 1931)	TPUT	Klie (1931)	
*T.* *serbani* (Danielopol, 1982)	TSER	Danielopol (1982)	
*T.* *sywulai* (Namiotko et al., 2004)	TSYW	Namiotko et al. (2004)	Duderina Jama, Croatia
*T.* *szoesci* (Farkas, 1958)	TSZO	Farkas (1958)	
*T.* *transylvanica* (Iepure et al., 2007)	TTRA	Iepure et al. (2007)	
*T.* *trigonela* (Klie, 1931)	TTRI	Klie (1931)	
Genus *Candona* Baird, 1845			
*C.* *candida* (O.F. Müller, 1776)	CCAN		Gdańsk, Łączyński Młyn, Lake Raduńskie Górne, Poland
*C.* *weltneri* Hartwig, 1899	CWEL		Lake Nierzostwo, Poland

For the number of haplotypes and GenBank Accession numbers of the obtained *COI* sequences see Table S1.

When our new species *Pseudocandona* *movilaensis* sp. nov. was analyzed using the existing CAP model to classify this species into one of the three existing groups specified above, it was clearly located within the cloud of the triangular *Typhlocypris* species, close to *T.* *marmonieri* (Fig. 3) with the distance to the centroid of this group 0.029, compared with distances of 0.399 and 0.352 to the centroids of the groups of *Pseudocandona* gr. *compressa* and *P*. gr. *rostrata*, respectively. Although uneven number of species was included in the three studied groups (Table 1), the distance-based test for homogeneity of multivariate dispersion (PERMDISP) showed no statistically significant differences (F = 2.557; P(perm) = 0.170) in the within-group multivariate dispersion among the three groups. To conclude, based on the valve shape in lateral view *Pseudocandona* *movilaensis* sp. nov. resembles to a great extent the stygobitic species of the genus *Typhlocypris*.

### Molecular phylogenetic analysis

**Figure 4:**
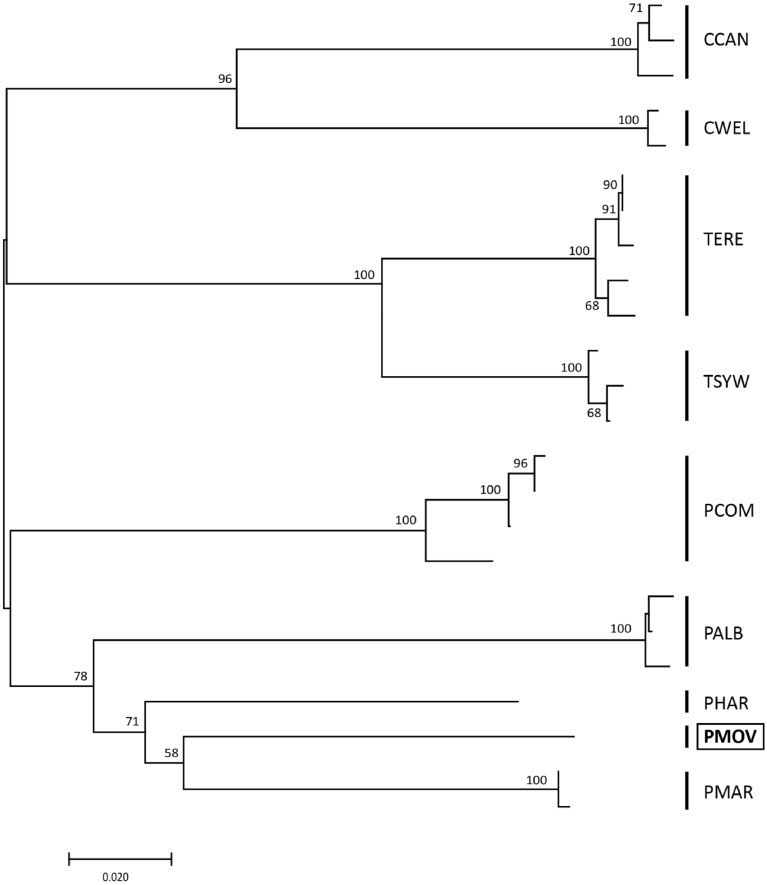
Neighbor-joining tree of the studied ostracod species based on the *COI* gene sequences (for species codes see Table 1). The distances were calculated with Kimura 2-parameter method. The numbers in front of the nodes indicate bootstrap support (1000 replicates, only values higher than 50% are presented).

In the NJ tree generated based on the haplotype COI data set (Table 1, Table S1), only the shallow branches were well-resolved (Fig. 4). The deep nodes remained poorly supported as the COI marker is unsuitable for the phylogenetic reconstructions of deep evolutionary histories. Nevertheless, focusing on well-supported terminal branches, our results showed that *Pseudocandona* *movilaensis* sp. nov. appeared to be closely affiliated to *Pseudocandona* species (Fig. 4). Furthermore, the new species is close to the clade formed by species of the *Pseudocandona* *rostrata* group (*P.* *marchica* and *P.* *hartwigi*) with the mean K2P pairwise genetic distances at the level of 0.15 (Table S2). The mean genetic distance between *P.* *movilaensis* sp. nov.and the *P.* *compressa* species-group (*P.* *albicans* and *P.* *compressa*) was 0.20, whereas between *P.* *movilaensis* sp. nov. and species of the genus *Typhlocypris* 0.24.

### Habitat characteristics, ecology and distribution

*Pseudocandona* *movilaensis* was reported from sulfidic thermal groundwaters (21 °C) characterized by slightly alkaline waters (pH of 7.2) and high concentrations of sulfide (0.25 mol dm^−3^), methane (0.02 mol dm^−3^) and ammonia (0.28 mol dm^−3^)^5^.

**Figure 5:**
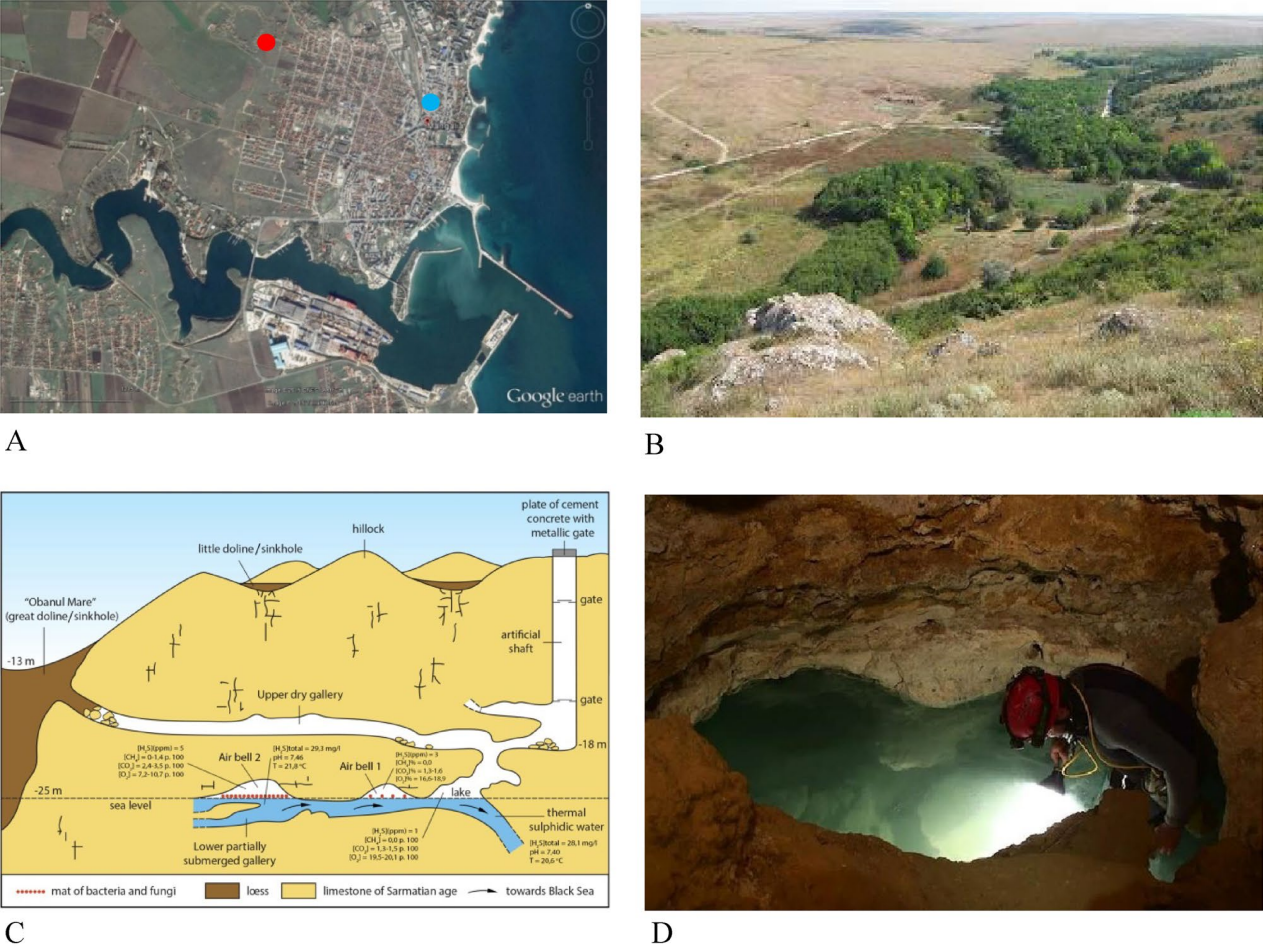
Location of Movile Cave near Mangalia (south eastern Romania). (**A**) Location of Movile Cave (red dot) and the hand-dug well in Mangalia village (blue dot). (**B**) Landscape around Movile Cave. (**C**) Profile of Movile Cave (map after Sarbu, 2000). (**D**) Lake Room from where the specimens were collected.

The species is known exclusively from the sulfide waters of the Movile Cave (SE Romania, 2 km from the Black Sea shore) in the Lake Room (of 1.5 m in diameter and the water depth of about 1 m) located at 18 m below the surface and representing the first 40 m long partially flooded gallery as well as from two hand-dug wells located at ca. 1 km from the cave^5,22^. The specimens from wells were only empty carapaces and it is assumed that the species may live in other sulfidic sites within the mesothermal aquifer from Mangalia or the presence of the carapaces in the well is the result of the passive transport with the groundwater flow (Fig. 5).

In situ live specimens in Lake Room were observed to live close to the wall and move downward to 10 cm to the bottom lake (which is almost hypoxic) and return to the surface after few seconds. They probably live at the redox interphase between the oxygenated and the cross-formational rising anoxic water (the top 10 cm of the Lake Room water), where the chemosynthetic sulfide-oxidizing bacteria thrive. The examination of live and dead specimens immediately after sampling revealed that all living specimens bear bacterial filaments on the shells, whereas none of the carapaces of dead animals presents these attachments.

## Discussion

### First taxonomic description of an ostracod from a SCE

Non-marine ostracods reported from sulfidic groundwaters are very rare, although non-marine ostracods generally thrive in a large array of extreme habitats, such as hot springs (with temperatures exceeding 50 °C), cold (up to freezing temperature), acidic (with pH as low as 3.4), and hypersaline waters (at salinities in excess of 100‰) as well as in temporary ponds prone to frequent complete drying or in deep groundwaters^35,36^.

As it stands, there are few well-documented SCE continental sites where ostracods are essential contributors to species diversity and an important functional group in the food web network. Among them are Movile Cave in Romania, Frasassi Cave in Italy, Ayalon Cave in Israel, and the more recently discovered Melissotrypa Cave in Greece^5,10,11,15^. Ostracods in SCE, however, are yet to be taxonomically studied to determine their species-specific adaptations to SCE or to investigate the environmental conditions in sulfidic waters that govern species spatial distribution.

### Homoplasy, phylogeny, troglomorphic features, and adaptation to subterranean realm

*P.* *movilaensis* sp. nov.has three distinctive morphological traits, which we consider homoplastic, i.e. gained or lost independently by species representing separate phylogenetic lineages: (1) triangular shape of the carapace and left valve in lateral view, (2) lack of so-called male bristles on the second antenna (setae t_2_ and t_3_ are not transformed in males into thick sensory bristles and remain similar to their counterparts in females), and (3) reduction of the posterior claw of the uropodal ramus in both sexes (with stronger reduction in males).

There is a striking and absorbing resemblance in general triangular carapace shape between several subterranean species belonging to various genera of the subfamily Candoninae. This triangular shape is one of the diagnostic traits (coupled with fine valve ornamentation and narrow inner lamellae in both adult and juvenile stages) of the exclusively subterranean genus *Typhlocypris*^37^, which shares this trait with some species of morphologically distinct genera containing species having mostly different (non-triangular) carapace shapes. Examples include subterranean *Fabaeformiscandona* *aemonae*, *Mixtacandona* *tabacarui*, *Schellencandona* *triquetra*, and *Candonopsis* *mareza* but also epigean species living in ancient lakes of Ohrid (e.g., *Neglecandona* *goricensis* and *N.* *litoralis*) and Baikal (e.g., *Baicalocandona* *navitarum* and *B.* *zenkevichi*)^38^. These ‘triangular’ species, however, can be morphologically easily distinguished from the lineage constituting the genus *Typhlocypris* based on differential diagnostic characters (of both carapace and limbs) of the genera to which they belong. *P.* *movilaensis* sp. nov. also possesses the carapace of triangular shape in lateral view (Fig. 1A,D), which suggests a close affinity with species of the genus *Typhlocypris* (Fig. 3). The only character of the valve morphology of *P.* *movilaensis* sp. nov. differing from species of the genus *Typhlocypris* are the slightly wider calcified inner lamella in female LV, amounting anteriorly to 10–11% of the valve length and being approximately 2.4× as wide posteriorly (in *Typhlocypris* usually it is ≤ 10% and < 2.0×, respectively). *P.* *movilaensis* sp. nov.shows a blend of morphological characters shared with species belonging to the genus *Typhlocypris* and to the *rostrata*-group of the genus *Pseudocandona* (see Diagnosis above and Description in Supplementary Materials). For these reasons, closer relationship of this ostracod either to *Typhlocypris* or *Pseudocandona* gr. *rostrata* based on morphological characters may be considered tenuous at best. The phylogenetic placement of *P.* *movilaensis* sp. nov. on the *COI* sequence tree (Fig. 4) and genetic distances with other studied species (Tables S2 and S3), however, supports hypothesis of its closer affinity with the species of the *rostrata*-group of the genus *Pseudocandona*, which typically develop carapaces of rectangular shape when viewed laterally. Thus, more distant relationship of *P.* *movilaensis* sp. nov. and *Typhlocypris* implies homoplastic evolution of the triangular carapace shape. The only species of the genus *Pseudocandona* with triangular carapace (except for some Baikalian species) is *P.* *punctata* known from lakes in Ohio^39^, but this species has an isolated position in the genus (with possible affinities with Baikalian candonids) and differs from *P.* *movilaensis* sp. nov. in ornamented valves, shape of male prehensile palps, straight (not curved) h_1_ seta on the cleaning leg, and the shape of lobes of hemipenis^40^. Although denser species sampling is needed for genetic data (but see results of the preliminary phylogenetic studies presented in^41^, where this species was initially marked as *Pseudocandona* sp. nov.), our new findings add to previous morphological evidence for morphological homoplasy of the triangular carapace shape among species of various genera of the subfamily Candoninae, further disassociating a polyphyletic group of ‘triangular’ Candoninae into different genera.

Typically, in the subfamily Candoninae, the second antenna (A2) is sexually dimorphic. In males, among other dimorphic traits, the penultimate segment is subdivided and bears the so-called male bristles, which play important prehensile and sensorial roles during courtship and copulation. These bristles are believed to be modified setae t_2_ and t_3_, which in females remain untransformed and are set on the undivided penultimate segment^42^. *P.* *movilaensis* sp. nov. has rare morphological combination, i.e., lacks male bristles but the penultimate segment of A2 is at least slightly separated by a thin chitinous septum. Within Candoninae, in some species or entire genera, male bristles are absent (with the penultimate segment divided or undivided), and it seems such cases are more frequent in subterranean lineages than in species inhabiting surface waters. Subterranean examples include all or most species of several endemic Nearctic genera of the tribe Cabralcandonini^43^; several Australian genera (e.g., *Leicandona* and all genera of the tribe Humphreyscandonini); Neotropical genus *Danielocandona*; Afrotropical genus *Namibcypris*; Palaearctic *Marococandona* and *Marmocandona*^44^ (and references therein); as well as some or single species of the genera *Mixtacandona*, *Trajancandona*, and *Typhlocypris*. Examples from surface waters are rarer and include some or single species of the genera *Baicalocandona*, *Candona*, *Cubacandona*, and *Paracandona* as well as few species of *Pseudocandona*. As for the latter genus, thus far, males of only four species have been known to lack male bristles, viz. European *P.* *insculpta* and *P.* *regnisnicolai* of the *compressa*-species group^45^, north American *P.* *punctata* of the *carribeana*-species group^40^, and Japanese *P.* *atmeta* of the *rostrata*-species group^46^. All these species (except for *P.* *punctata*, see above) have non-triangular carapace shape, clearly distinguishing them from *P.* *movilaensis* sp. nov. Our new species differs also from ‘triangular’ *Typhlocypris* *pretneri* (the single species lacking male bristles) in the morphology of the hemipenis and uropod. In any case, the lack of male bristles in different genera (or even tribes) within Candoninae indicates homoplastic evolution, implying that developmental transformation of t-setae into male bristles may be caused by recurrent mutations across not closely related taxa.

In the subfamily Candoninae, uropod commonly consists of two rod-shaped rami, each bearing distally two claws and two setae^42,44^. A number of various reductions of this chaetotaxic scheme have been described within separate genera and tribes. The common reductions include the absence of a posterior seta (e.g., some genera of the tribe Candonopsini) or reduction of size, transformation to seta, or complete lack of a posterior claw G_p_ (as e.g., in the genus *Meischcandona* or in several subterranean genera endemic to Australia). In some species, the uropodal ramus is strongly reduced with only one apical claw or seta (e.g., some genera of the tribe Cabralcandonini^43^ and the tribe Namibcypridini^47^) or even the ramus is reduced to a flagellum without any setae or claws (as in *Cabralcandona*)^43^. Beyond doubt, simplification of the uropodal ramus has occurred several times within the subfamily Candoninae, and if the similarity in the form of the caudal ramus exists in different lineages, it presents another example of homoplasy, which may create difficulties in phylogenetic analysis. In the four closely related genera: *Pseudocandona,* *Typhlocypris*, *Schellencandona*, and *Marmocandona*, the uropodal ramus is well-developed, with two claws and two setae. To our knowledge, *P.* *movilaensis* sp. nov. is unique in having reduced G_p_ claw in both sexes. The only other species of *Pseudocandona* with reduced G_p_, but only in males, is *P.* *marchica*, which can be easily distinguished from our new species by having a non-triangular carapace shape and well-developed male bristles^42^.

Although traditionally homoplasies are considered to be caused by convergence (when arising by different developmental pathways) or parallelism (if similar developmental mechanisms are involved)^48^, some evolutionary biologists argue that convergent and parallel evolution are difficult to distinguish as there is a continuum between these, and thus, propose to use a single term—convergent evolution^49^. Nevertheless, at this stage, it is entirely speculative if the three above-mentioned homoplastic traits have evolved independently in *P.* *movilaensis* sp. nov.

Stygobitic cavernous crustaceans belonging to different phylogenetic groups evolve independently with similar suits of traits termed troglomorphic^50^. For example, several amphipods and isopods inhabiting cave waters show increased appendage length or setation and advanced development of chemo-sensorial organs^29^. As presented above, the three homoplastic characters of *P.* *movilaensis* sp. nov. can be also considered troglomorphic. We hypothesize that at least two of these traits (lack of male bristles and triangularly-shaped carapace) may have resulted from paedomorphosis, a well-known heterochronic evolutionary process of the retention of youthful ancestral features by adult descendants^51^. There are two distinct processes explaining paedomorphosis: acceleration of sexual maturation relative to the rest of development (progenesis) and retardation of somatic development with respect to the onset of reproductive activity (neoteny). We believe that paedomorphic characters of *P.* *movilaensis* sp. nov. results from neoteny rather than progenesis. The pressures that cause either of these types of paedomorphic evolution remain unclear. Future investigations should address this problem through in situ and/or laboratory studies on developmental changes in morphology and on the life history of our new species.

The question arises whether the troglomorphic characters of *P.* *movilaensis* sp. nov. are adaptations to general cavernous conditions or specifically to SCE. Reduced posterior claw G_p_ on the uropodal ramus has probably no adaptive value because the energetic expense connected with the reinforcement of that seta into a claw would be minimal. It is also hard to deduce if uropod, with one ‘normally’ developed long anterior claw and short G_p_, could be more effectively used to glide over the mat of sulfidic bacteria compared to the use of typical Candoninae uropod with two long claws. At this stage, it is also entirely speculative if ‘immaturity’ of retarded development of the t_2_ and t_3_ setae (resulting in the absence of chemo-sensorial male bristles) has been caused by any specific selection for such morphology, which should then have adaptive advantages in SCE. Unfortunately, clear comparative experimental data on mating or pre-copulatory behaviour of species with and without developed male bristles are so far lacking, thus, preventing an opportunity to contrast possible differences and function of this sexually dimorphic feature within the subfamily Candoninae.

The carapace of ostracods acts as an interface between the organism and environment and is more likely to be subjected to selection pressures and to have an adaptive value^52^. The adaptive significance of the valve shape among the true subterranean ostracods is still a debated issue^53^. If the evolutionary process is not driven by the selective pressure of cave conditions, ‘triangularization’ of the ostracod carapace shape may start either outside the cave (being already present in the ancestor or gained during the colonization of the cavernous environment) or inside the cave (gained after successful colonization of the subterranean realm). We hypothesize that triangular carapace shape may be an adaptive feature selected under environmental conditions in caves, where underdevelopment of the postero–dorsal section of the carapace may provide energetic solution to the oligotrophic cave conditions (less material needed, see below) coupled with low reproduction rate (less space needed for lower number of eggs).

### Engineering construction of the carapace

**Figure 6:**
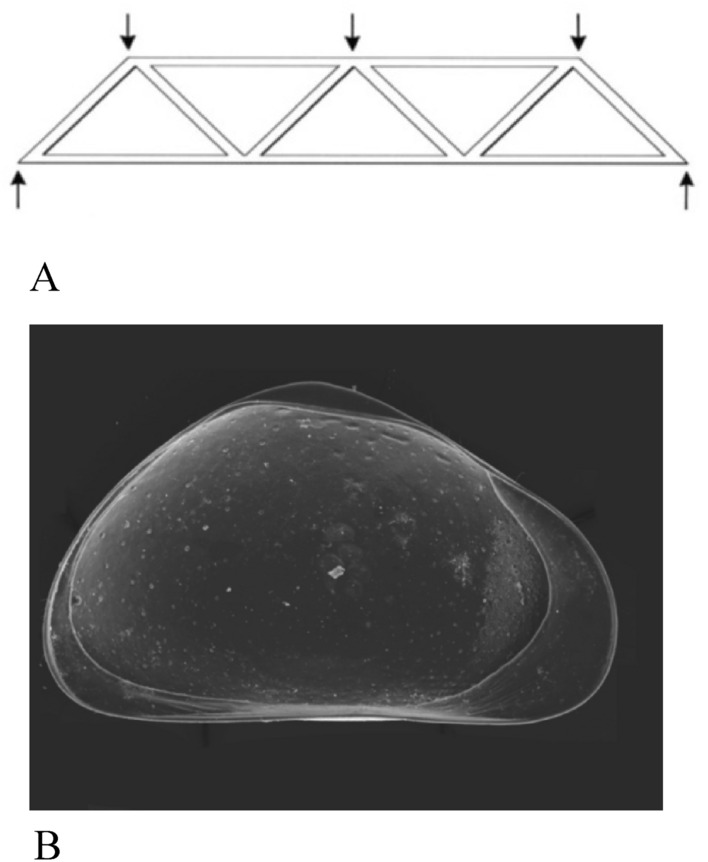
(**A**) Architectural representation of the triangular shape of ostracod carapace. (**B**) Female left valve in lateral view of *Pseudocandona* *movilaensis* sp. nov.

The carapace in ostracods has functional implications, and it is viewed as an efficient ‘engineering construction’ adopting a shape and a structure design according to the environmental conditions in which the species lives with the use of minimal amounts of material^54^. The first author advancing the idea that the ostracod carapace ‘is a static frame structure with a shape, which, during evolution, can be deformed following specific rules’ was Benson^55^. He stated that a hint to understand the solutions adopted by ostracods to obtain the most advantageous carapace shape can be traced by making analogies with the techniques used in architecture constructions. Hence, the ostracod carapace is seen as having similar design to a dome with a double walled cupola with the exterior part being thicker and more resistant and the internal one thin (Fig. 6). Later on, Danielopol^52^ advanced the idea that the triangular shape of the ostracod valves is a benefit and fitness solution for species thriving in subterranean environment. The triangular shape of carapace is viewed in a similar way as a tripod, wherein the weight is distributed more efficiently among the three faces (Fig. 6). In agreement with the principles of geometry and mechanics, it is well-known that a triangular shape structure in general has two advantages: (1) deformation is more difficult and is able to balance the stretching and compressive forces inside the structure and (2) is less costly as it requires less material to make the three sides of the triangle.

The triangular shape of the carapace can also have an ecological meaning. In an environment with a high concentration of sulphide and methane, the species must take protective measures against the diffusion of these elements from water into the body^56^. For example, marine ostracods from thermal vents have a waterproof shell^57^. Moreover, an appropriate shell shape can also help the animal to reduce the surface area and volume so that diffusion of toxic elements is minimal. Hence, the triangular valve shape remarked in *P.* *movilaensis* sp. nov. vs. the typical rectangular shape of the *Pseudocandona* species from the *rostrata*-group (even taking into account the simplification of the three-dimension-model to 2D) may offer a selective advantage for the species that developed or retained this solution, which was already present in its ancestor.

### Ancientness

Some insight into the early evolution of the triangular-shaped Candoninae lineages can be gleaned from fossil record. The oldest records of triangular Candoninae assigned to the phylogenetic lineage of the genus *Typhlocypris* can be traced to *T.* *pechelbronnensis* from Oligocene to Early Miocene deposits in eastern France and western Germany as well as *T.* *roaixensis* and *T.* *ratisbonensis* from Miocene in southern France and Czech Republic. These three species were recorded from epigean fresh or brackish water paleo-habitats and may be hypothetical ancestors of the *Typhlocypris* lineage^37,58^. The triangular shape of the valves in lateral view, which resembles that of various living triangular stygobitic European species, has been also observed in a number of fossil species of Late Miocene to Quaternary deposits in Europe and Western Asia, e.g., *Candona* (*Typhlocypris*) aff. *eremita* from the Late Miocene (ca. 11.6 Mya) in (paleo) Lake Pannon^59^ or *Caspiocypris* *schneiderae* and *Caspiocypris* *ola* from the Pliocene and Quaternary deposits in Azerbaijan^60^. Some of these species may represent either the lineage of *Typhlocypris* or are ancestors of other recent triangular European species, including our new *P.* *movilaensis* In order to account for such relationships forming a basis of taxonomical categorization, however, further comparative morphological studies are needed using original material. The ancientness of the potential ancestors of *P.* *movilaensis* sp. nov. may be further deduced with the onset of the underground system of the Movile Cave dating back to the Late Miocene (ca. 12.5 Mya)^5^.

Finally, it is noteworthy to mention the recent studies on fossil ostracods from Late Pleistocene sediments (ca. 15,000 years ago) in the north-west part of the Black Sea (ca. 4 m depth)^61^. Furthermore, in the sediments deposited under oxygenated bottom-water conditions, abundant populations of Candoninae (a group consisting of mostly freshwater species) were found, with few specimens showing triangular left valve. The presence of Candoninae in marine sediments of the Black Sea is associated with a shift in geochemical settings and a sharp rise in the carbonate content about 15,000 years ago in the area. This suggests an increase of melt-water pulses that were discharged into the Black Sea basin from the Eurasian region and a passive transport of freshwater ostracods into the sea.

## Conclusions

This is the first taxonomic description of a freshwater ostracod species living exclusively in sulfidic cave waters. We assume that the triangular valve shape in the newly described cave species *P.* *movilaensis* sp. nov. is a paedomorphic trait, which could be advantageous in the groundwater environment. We further assume that phenotypic similarity in the valve shape of *P.* *movilaensis* sp. nov. with the stygobite species of the genus *Typhlocypris* (as well as with other triangular-shaped stygobitic species of other Candoninae genera) is a homoplasy caused by convergent or parallel evolution, attributable to the similar environmental constraints that need to be overcome to survive in the extreme subterranean realm.

The position of the new species within the *rostrata* group of the genus *Pseudocandona*, as indicated by the *COI* phylogeny, also corroborates the homoplastic nature of other traits that are shared with species morphologically assigned to separate lineages. These results suggest that some traditional characters used to unite certain non-marine ostracods evolved more than once, often obscuring their true phylogeny. Our findings of a new non-marine ostracod thriving in rich sulfidic groundwaters open the pathway to boost further studies on morphological, physiological, and metabolic adaptations to this specific type of waters; identify if the ecological plasticity of certain species that enable them to live in the toxic environment; and if there is a congruence in adaptive strategies adopted by ostracod species from continental groundwater vs. thermal vents. Movile Cave is in a coastal aquifer, which is affected by anthropogenic and natural alterations. These impacts may have implications for management of groundwater and fauna from other sea–ocean–freshwater groundwater connected systems. Hence, further studies may be oriented on the anthropogenic impact (e.g., overexploitation and contamination of the aquifer) and climate change effects on species and habitats.

## Methods

### Sampling and description

More than 40 specimens of ostracod crustaceans were collected from the sulfidic waters of the Lake Room in Movile Cave, Romania (43°49.611' N, 28°33.684' E)^5^ in three sampling campaigns in 1990 (May), 2012 (April, September) and 2015 (May) by Serban Sarbu (Fig. 5). Specimens were taken from the top 10 cm of the Lake Room walls with a pipette and preserved in 90% ethanol. There were no replicates taken since the purpose of the study was taxonomic. Samples were taken once at each sampling time until the number of specimens were attained for the scope of our study.

Lake Room represents the piezometric level of the mesothermal sulfidic aquifer of Mangalia which occupies a surface of approximately 50–100 km^2^. Lake Room is located at 18 m depth below the surface, has a diameter of 1.5 m and the water depth is about 1 m^6,22^. The water at the surface is relatively stagnant while a slow groundwater flow is present at depths over 1 m^22^. The physico-chemical parameters of the water are constant, and no fluctuations have been registered during each sampling campaign (H_2_S = 30 mg/L; pH  7.2 (average), temperature = 19–21 °C).

The ostracod specimens were always present in the Lake Room irrespective of the sampling season. No populational study has been performed so far, and hence, there is currently no estimation of the population size.

The Appendages were dissected and mounted in glycerol on the glass slides, whereas disarticulated carapace valves of the dissected specimens were stored dry in micropaleontological slides. Undissected specimens are stored in 90% ethanol. Appendages and valves were drawn with the aid of a camera lucida. Scanning electron images of valves of selected specimens were obtained with a JEOL SM-31010 Scanning Electron Microscope at the Zoological Museum, University of Copenhagen, Denmark.

### Localization of the cave

The map used in Fig. 5A was created using Google Earth Version 9.167.0.0 (25 July 2022), Movile Cave, Romania; 43°49′29′′ N, 28°33′40′′ E, 23 m a.s.l. DigitalGlobe 2022. http://www.earth.google.com [25 July 2022].

### Geometric morphometry analyses

For geometric morphometrics, the left female valve of *Pseudocandona* *movilaensis* was photographed in external view using an Olympus light microscope and an Olympus digital camera and processed by Adobe Photoshop^62^. To compare the valve shape of a new species with other 25 species of the genera *Pseudocandona* and *Typhlocypris*, we used published descriptions and illustrations (Table 1). The obtained outlines were digitized with TpsDig2 software, version 1.37 for further morphometric analyses^63^. The geometric analyses of the outlines were performed using the Linhart B-spline algorithm in Morphomatica v. 1.6 using 32 control points^64^. The obtained Mean Delta Square distances were used as morphological disparities between the obtained valve outlines. The distance matrix was subsequently used to discriminate between species belonging to three groups: A) 13 species of the genus *Typhlocypris*, B) seven species of the *rostrata*-group of the genus *Pseudocandona*, and C) five species of the *compressa*-group of the genus *Pseudocandona* (Table 1). We used Canonical Analysis of Principal Coordinates (CAP) implemented in the PERMANOVA+ add-on to PRIMER v7 software to predict the genus/species-group to which individual species belong based on the valve shape and to diagnose misclassification error^65^. Having the CAP model, *Pseudocandona* *movilaensis* was placed onto the obtained canonical axes to classify this species into one of the three existing groups specified above. In addition, we performed a test of the null hypothesis of no differences in the within-group multivariate dispersion among the three groups by PERMDISP routine in PERMANOVA+ ^65^.

### Molecular phylogenetic analysis

Genomic DNA was extracted from 38 specimens, representing nine selected ostracod species of the subfamily Candoninae (Table 1, Table S1). Details of the DNA extraction, amplification and sequencing procedure were described previously^41^. The DNA barcoding fragment of Cytochrome-c-Oxidase subunit I gene (COI)^66^ was amplified using standard primers LCO1490/HCO2198^67^. The cleaned PCR products were directly sequenced in both directions with the BigDyeTM terminator cycle sequencing method using the PCR primers by Macrogen Inc. BLAST^68^ searches against the non-redundant database of the National Center for Biotechnology Information (NCBI) were performed to verify the sequence similarity of the amplified region. The 38 newly obtained sequences of COI were aligned with MAFFTv7.405^69^ using the automatic algorithm and trimmed in GENEIOUS 10.0.2 (available at: http://www.geneious.com) leading to 567 bp long alignment. The number of unique haplotypes was calculated in DnaSp^70^. As a potential outgroup, three haplotypes of *Candona* *candida* and two haplotypes of *Candona* *weltneri* were used. All sequences were deposited in GenBank with the accession numbers (Table S1).

Mean genetic distances under the Kimura 2-parameter model (*K2p*)^71^ between COI data set obtained from the nine ostracod species were calculated in MEGA X 10.0.3^72^ (Tables S2 and S3). For graphic presentation of the relationships among the studied species, Neighbour-Joining (NJ) tree for COI data was generated using K2P distances with 1000 bootstrap replicates in MEGA.

## Supplementary Information

Supplementary Information.

## Data Availability

The type series of the new species are available in the National Museum of Nature and Science, Tsukuba (NSMT) (*Pseudocandona* *movilaensis* sp. nov.). The sequence datasets generated and/or analyzed during the current study are available in The International Nucleotide Sequence Database Collaboration (INSDC) repository, https://www.ncbi.nlm.nih.gov/nuccore/MN013132. Species locality and accession number for the specimens of the new species are presented in Supplementary material Table S1.

## References

[1] Engels AE (2007). Observations on the biodiversity of sulfidic karst habitats. J. Cave Karst Stud..

[2] Mulec J, Engels AS (2019). Karst spring microbial mat microeukaryotic diversity differs across an oxygen-sulphide ecocline and reveals potential for novel taxa discovery. Acta Carsol..

[3] Mulec J, Ooarga-Mulec A, Schiller E, Persoiu A, Holko L, Šebela S (2015). Assessment of the physical environment of epigean invertebrates in a unique habitat: The case of a karst sulfidic spring, Slovenia. Ecohydrology.

[4] Brazelton W (2017). Hydrothermal vents. Curr. Biol..

[5] Sarbu, S. M. Movile Cave: A chemoautotrophically based groundwater ecosystem. in *Subterranean**Ecosystems* (Wilken, H., Culver, D.C. & Humphreys, W.F. eds.). 319–343 (Elsevier, 2000).

[6] Brad T, Iepure S, Sarbu S (2021). The chemoautotrophically based movile cave groundwater ecosystem, a hotspot of subterranean biodiversity. Diversity.

[7] Schreier, J. E. & Lutz, R. A. Hydrothermal vent biota. in *Encyclopedia**of**Ocean**Sciences*. 3rd Edn. 308–319 (Academic Press, 2019).

[8] Kalenitchenko D, Le Bris N, Dadaglio L, Peru E, Besserer A, Galand PE (2018). Bacteria alone establish the chemical basis of the wood-fall chemosynthetic ecosystem in the deep-sea. ISME J..

[9] Hourdez, S. & Jollivet, D. Metazoan adaptation to deep-sea hydrothermal vents. in *Life**in**Extreme**Environments* (di Prisco, G., Howell, G., Edwards, G. M., Elster, J. & Huiskes H. L. eds.). 42–68 (Cambridge University Press, 2020).

[10] Por FD, Dimentman Ch, Frumkin A, Naaman I (2013). Animal life in the chemoautotrophic ecosystem of the hypogenic groundwater cave of Ayyalon (Israel): A summing up. Nat. Sci..

[11] Peterson DE, Danielopol D, Finger K, Iepure S, Mariani S, Montanari A, Namiotko T (2013). Reconnaissance of ostracode assemblages in the Frasassi Caves, the adjacent sulfidic spring and the Sentino River in the northeastern Apennines (Marche region, Italy). Cave Karst Sci..

[12] Flot (2010). Unsuspected diversity of Niphargus amphipods in the chemoautotrophic cave ecosystem of Frasassi, central Italy. BMC Evol. Biol..

[13] Bauermeister J, Ramette A, Dattagupta S (2012). Repeatedly evolved host-specific ectosymbioses between sulfur-oxidizing bacteria and amphipods living in a cave ecosystem. PLoS ONE.

[14] Galassi DMP, Fiasca B, Di Lorenzo T (2017). Groundwater biodiversity in a chemoautotrophic cave ecosystem: How geochemistry regulates microcrustacean community structure. Aquat. Ecol..

[15] Popa I (2019). Rich and diverse subterranean invertebrate communities inhabiting Melissoreypa cave in central Greece. Trav. Institut de Spéol. “Émile Racovitza”.

[16] Chen Y, Wu L, Boden R (2009). Life without light: Microbial diversity and evidence of sulfur- and ammonium-based chemolithotrophy in Movile Cave. ISME J..

[17] Hutchens E, Radajewski S, Dumont MG, McDonald IR, Murrell JC (2004). Analysis of methanotrophic bacteria in Movile Cave by stable isotope probing. Environ. Microbiol..

[18] Flot JF (2014). *Niphargus-Thiothrix* associations may be widespread in sulphidic groundwater ecosystems: evidence from southeastern Romania. Mol. Ecol..

[19] Kumaresan D (2014). Microbiology of Movile Cave—A chemolithoautotrophic ecosystem. Geomicrobiology.

[20] Sarbu SM, Kane TC, Kinkle BK (1996). A chemoautotrophically based cave ecosystem. Science.

[21] Dahl TW, Siggaard-Andersen ML, Schovsbo NH (2019). Brief oxygenation events in locally anoxic oceans during the Cambrian solves the animal breathing paradox. Sci. Rep..

[22] Sarbu, S. M. & Popa, R. A unique chemoautotrophically based cave ecosystem. in *The**Natural**History**of**Biospeleology* (Camacho, A.I. ed.). 637–666 (National Museum of Natural History, 1992).

[23] Boston PJ (1999). The search for extremophiles on Earth and beyond: What is extreme here may be just business-as-usual elsewhere. Ad Astra (Washington, D.C.).

[24] Jahn A, Janas U, Theede H, Szaniawska A (1997). Significance of body size in sulphide detoxification in the Baltic Clam Macoma Balthica (Bivalvia, Tellinidae) in the Gulf of Gdańsk. Mar. Ecol. Prog. Ser..

[25] Hourdez S, Lallier FH (2007). Adaptations to hypoxia in hydrothermal-vent and cold-seep invertebrates. Rev. Environ. Sci. Biotechnol..

[26] Theede H, Ponat A, Hiroki K (1969). Studies on the resistance of marine bottom invertebrates to oxygen-deficiency and hydrogen sulphide. Mar. Biol..

[27] Vismann R (1991). Sulfide tolerance: physiological mechanisms and ecological implications. Ophella.

[28] Bagarinao T (1992). Sulfide as an environmental factor and toxicant: tolerance and adaptations in aquatic organisms. Aquat. Toxicol..

[29] Culver, D. C. & Pipan, T. Adaptations to subterranean life. in *The**Biology**of**Caves**and**Other**Subterranean**Ecosystems* (Culver D.C. & Pipan T. eds.). (Oxford University Press, 2019).

[30] Smith, A. J., Horne, D. J. Ecology of marine, marginal marine and non-marine Ostracodes. in *The**Ostracoda:**Applications**in**Quaternary**Research.**Geophysical**Monograph* (Holmes, J.A. *et**al*. eds.). Vol. 131. 37–64 (2002).

[31] Horne DJ (2003). Key events in the ecological radiations of the Ostracoda. Paleont. Soc. Pap..

[32] Horne DJ (2005). Homology and homoeomorphy in ostracod limbs. Hydrobiology.

[33] Wake DB (1991). Homoplasy: the result of natural selection, or evidence of design limitations?. Am. Nat..

[34] Schallreuter, R. E. Homeomorphy, phylogeny and natural classification: case studies involving Palaeozoic ostracods. in *Evolutionary**Biology**of**Ostracoda:**Its**Fundamentals**and**Applications* (Hanai, T., Ikeya, N. & Ishizaki, K. eds.). 1041–1049 (Elsevier, 1988).

[35] Griffiths, H. I. & Holmes, J. A. Non-marine ostracods and quaternary palaeoenvironments. in *Quaternary**Research**Association.**Technical**Guide* 8. (2000).

[36] Laprida C, Diaz A, Ratto N (2006). Ostracods (Crustacea) from thermal waters, southern Altiplano, Argentina. Micropaleontology.

[37] Namiotko T, Danielopol DL, Meisch C, Gross M, Mori N (2014). Redefinition of the genus *Typhlocypris* Vejdovsky, 1882 (Ostracoda, Candonidae). Crustaceana.

[38] Karanovic I, Sitnikova TY (2017). Morphological and molecular diversity of Lake Baikal candonid ostracods, with description of a new genus. ZooKeys.

[39] Furtos N (1933). The Ostracoda of Ohio. Bull. Ohio Biol. Surv..

[40] Karanovic I (2006). Recent Candoninae (Crustacea, Ostracoda, Candonidae) of North America. Rec. West. Aust. Mus. Suppl..

[41] Wysocka A, Kilikowska A, Mori N, Iepure S, Kijowska M, Namiotko T (2019). Monophyletic status of European morphogenera of the subfamily Candoninae Kaufmann, 1900 (Ostracoda: Candonidae) in relation to their mtDNA phylogenies. J. Crust. Biol..

[42] Meisch, C. Freshwater Ostracoda of Western and Central Europe. in *Süßwasserfauna* *von**Mitteleuropa**8/3* (Schwoerbel, J. & Zwick, P. eds.). 522 (Spektrum Akademischer, Gustav Fischer, 2000).

[43] Külköylüoğlu O, Yavuzatmaca M, Akdemir D, Yılmaz O, Çelen E, Dere S, Dalkıran N (2019). Correlational patterns of species diversity, swimming ability and ecological tolerance of non-marine ostracoda (Crustacea) with different reproductive modes in shallow water bodies of Ağrı region (Turkey). J. Freshw. Ecol..

[44] Karanovic I, Lee W (2012). A review of candonid ostracods (Crustacea: Ostracoda: Podocopida) from East Asia, with descriptions of five new species from South Korea. Zootaxa.

[45] Karanovic I, Petkovski TK (1999). Two new species of the subfamily Candoninae (Ostracoda) from Montenegro (SE Europe). Crustaceana.

[46] Smith RJ, Takahiro K (2015). Four new species of the subfamily Candoninae (Crustacea, Ostracoda) from freshwater habitats in Japan. Eur. J. Taxon..

[47] Martens K, Horne DJ (2000). Preface: Ostracoda and the four pillars of evolutionary wisdom. Hydrobiology.

[48] Hodin J (2000). Plasticity and constrains in development and evolution. J. Exp. Zool..

[49] Arendt J, Reznick D (2008). Convergence and parallelism reconsidered: what we learned about the genetics of adaptation?. Trends Ecol. Evol..

[50] Howarth F.G. & Moldovan O.T. The ecological classification of cave animals and their adaptations. in *Cave**Ecology* (Moldovan, O.T., Kováč L. & Halse S. eds.). 41–67 (Springer Link, 2018).

[51] Christiansen K, Culver DC, White WB (2005). Morphological adaptations. Encyclopedia of Caves.

[52] Danielopol, D. L., Olteanu, R. & Lete, C. Carapace morphology of *Cytherissa**lacustris* (Cytherideidae): Its interest for the systematics and the phylogeny of the group. in *Cytherissa**(Ostracoda),**The**Drosophila**of**Paleolimnology*. (Danielopol, D.L., Carbonel, P. & Colin, J.-P. eds). 47–48, 27–53 (Bull. l’Inst. Géol. Bassin Aquitaine, 1990).

[53] Rouch R, Danielopol DL (1987). L´origine de la faune acuatique souterraine entre le paradigme et du refuge et le modele de la colonization active. Stygologia.

[54] Benson RA (2003). Biomechanical theory of Ostracode carapace morphology. Paleontol. Soc. Pap..

[55] Benson, R. H. Morphologic stability in Ostracoda. in (Swain, E. M. Ed.) *Biology**and**Paleobiology**of"**Osrracoda.**Proceedings**of**the**4th**meeting**of**the**Ostracod**workers**in**Newark**(Delaware)**1972*. Vol. 65(282). 13~i6 (Bulletins American Paleontology/Paleontological Research Institution, 1975).

[56] Por F (2014). Sulfide shrimp? Observations on the concealed life history of the Thermosbaenacea (Crustacea). Subt. Biol..

[57] Tanaka H, Yasuhara M (2016). A new deep-sea hydrothermal vent species of Ostracoda (Crustacea) from the Western Pacific: Implications for adaptation, endemism, and dispersal of ostracodes in chemosynthetic systems. Zool. Sci..

[58] Iepure S, Namiotko T, Danielopol DL (2007). Evolutionary and taxonomic aspects within the species group *Pseudocandona*
*eremita* Vejdovský (Ostracoda, Candonidae). Hydrobiologia.

[59] Gross M (2004). Zur Ostracodenfauna (Crustacea), Paläoökologie und Stratigraphie der Tongrube Mataschen (Unter-Pannonium, Steirisches Becken, Österreich). Joannea Geol. Paläontol..

[60] Agalarova, D. A., Kadyrova, Z. K. & Kulieva, S. A. *Ostracoda**from**Pliocene**and**Postpliocene**Deposits**of**Azerbaijan*. 1–420 (Azerbaijan State Publishers, 1961) (**in Russian**).

[61] Boomer I, Guichard F, Lericolais G (2010). Late Pleistocene to recent ostracod assemblages from the western Black Sea. J. Micropalaeontol..

[62] Strake A, Danielopol DL, Neubauer W (2008). Comparative study of *Candona*
*neglecta* valves from the shallow and deep sites of Lake Mondsee. Ber. Inst. Erdwissenschaften K.F. Univ. Graz.

[63] Brauneis, W., Linhart, J., Stracke, A., Danielopol, D.L., Neubauer, W. & Baltanás, A. *Morphomatica**(Version**1.6)**User**Manual/Tutorial**Mondsee**User**Manual/Tutorial.* 1–82 (Limnological Institute, Austrian Academy of Sciences, 2006).

[64] Iepure S, Namiotko T, Danielopol DL (2008). Morphological diversity and microevolutionary aspects of the lineage *Cryptocandona*
*vavrai* Kaufmann 1900 (Ostracods Candon-inae). Ann. Limnol. Int. J. Limnol..

[65] Anderson, M.J., Gorley R.N. & Clarke K.R. *PERMANOVA+**for**PRIMER:**Guide**to**Software**and**Statistical**Methods*. (PRIMER-E, 2008).

[66] Hebert PD, Ratnasingham S, de Waard JR (2003). Barcoding animal life: Cytochrome c oxidase subunit 1 divergences among closely related species. Proc. R. Soc. B.

[67] Folmer O, Black M, Hoeh W, Lutz R, Vrijenhoek R (1994). DNA primers for amplification of mitochondrial cytochrome c oxidase subunit I from diverse metazoan invertebrates. Mol. Mar. Biol. Biotechnol..

[68] Altshul SF, Gish W, Miller W, Myers EW, Lipman DJ (1990). Basic local alignment search tool. Mol. Biol..

[69] Katoh K, Misawa K, Kuma K, Miyata T (2002). MAFFT: A novel method for rapid multiple sequence alignment based on fast Fourier transform. Nucleic Acids Res..

[70] Librado P, Rozas J (2009). DnaSP v5: A software for comprehensive analysis of DNA polymorphism data. Bioinformatics.

[71] Kimura M (1980). A simple method for estimating evolutionary rates of base substitutions through comparative studies of nucleotide sequences. J. Mol. Evol..

[72] Kumar S, Stecher G, Li M, Knyaz C, Tamura K (2018). MEGA X: Molecular evolutionary genetics analysis across computing platforms. Mol. Biol. Evol..

