# Supplementary material for: A new extremophile ostracod crustacean from the Movile Cave sulfidic chemoautotrophic ecosystem in Romania
Source: Sci Rep. 2023 Apr 14;13:6112. doi: 10.1038/s41598-023-32573-w (PMC10104858; doi:10.1038/s41598-023-32573-w)

SUPPLEMENTARY MATERIALS

Sanda Iepure, Anna Wysocka, Serban M. Sarbu, Michalina Kijowska, Tadeusz Namiotko

Extremophile at a glance: ostracod crustacean from a chemoautotrophic sulfidic cave ecosystem

Description of a new species *Pseudocandona movilaensis* sp. nov.

Carapace medium-sized with triangular lateral view and the greatest height at or just behind mid-length (at 50-60% of the length). Holotype female left valve (LV): length (L) = 1.1 mm, height (H) = 0.59 mm, H/L = 0.54. Carapace measurements of female paratypes: L = 0.98-1.05 mm (mean ± standard deviation SD = 1.006 ± 0.021, N = 9), H = 0.58-0.59 mm (mean ± SD = 0.582 ± 0.004, N = 9), H/L = 0.56-0.59 (mean ± SD = 0.579 ± 0.009, N = 9). LV overlaps right valve (RV) at both extremities. Female LV (Fig. 1A, D, Fig. S1A) relatively high (H/L = 0.53) and triangular in lateral view with greatest H located behind mid-length (54% of valve L). Dorsal margin of LV roundly arched with distinctive hump that overlaps RV. Ventral margin of LV slightly concave. Anterior and posterior margins infracurvate (i.e., unevenly curved with apex below mid-length), posterior one being more inflated. Calcified inner lamella not evenly curved, slightly wider at the antero-ventral and postero-ventral margins, at the anterior end of LV amounting to c. 10-11% of the valve L and ca. 2.4× as wide as posteriorly. Female RV (Fig. 1B-C, Fig. S1B) more trapezoidal in outline than the LV and lower in height, with the greatest H situated at 58% of L and with almost straight and then slightly concave antero-dorsal margin. Central muscle scar arrangement typical of the subfamily Candoninae (Fig. 1C). Valve surface smooth with fine ornamentation (shallow pits) only on the anterior and posterior margins, and covered with short and stiff setae on the entire area. Colour white.

Sexual dimorphism in size and shape of the carapace weakly expressed, male carapace slightly larger. Left male valve (Fig. 1E, Fig. S1C) similar to that of female (L = 1.18 mm) but slightly higher (H = 0.61 mm) with H/L = 0.52 and less triangular with postero-dorsal margin more rounded. RV (Fig. 1F) L = 1.21 mm, H = 0.61, H/L = 0.50. Carapace measurements of other male paratype L = 1.02 mm, H = 0.60 mm, H/L = 0.59.

Juvenile carapaces and valves of the last four juvenile stages (A-1 to A-4) are presented in Fig. S2. Carapace of A-1 juvenile viewed dorsally moderately compressed with greatest W inferior to 1/2 L and situated just behind mid-length (56-57% of L). Anterior end

slightly beak-shaped, posterior ends slightly pointed. Valve surface of A-3 juvenile with reticulate ornamentation.

Antennule A1 (Fig. S3A) with total of seven articulated segments. First segment bearing two long setae on dorsal margin and two long setae on ventral-distal corner. Second and third segments each with one dorsal-apical seta almost twice the length of the segments. Fourth and fifth segments each with two long dorsal-apical setae and one short ventral-apical seta. Penultimate segment with one short and three medium-length setae distally. Terminal segment with two long setae, one short seta and aesthetasc y_a as long as the terminal segment.

Female antenna A2 (Fig. 2A) with protopodite bearing reduced exopodite and 3-segmented endopodite. Protopodite typically with one long smooth basal seta. Exopodial plate with two very short setae and one long thin seta longer than the first endopodial segment (EI). Aesthetasc Y equalling c. 0.37 of EI (the sensory part of this organ represents 45% of its L). Setae t_1-t_4 short, representing 0.31-0.34 EI length. Relative lengths of claws, some setae and the remaining aesthetascs as compared with the L of EI as: G_1 (claw) = 0.96, G_2 (claw) = 0.57, G_3 (claw) = 1, z_1 = 0.15 (seta like), z_2 and z_3 (setae) = c. 0.17, G_M (claw) = 0.77, G_m (claw) = 0.36; y_1 = 0.1, y_2 = 0.1 and y_3 = 0.31 (sensory part hardly visible). Claw G_1 about 1.6 and claw G_M about 1.3 length of the anterior margin of combined endopodial podomeres EII and EIII.

Male antenna (Fig. 2B, Fig. S4A) with 3-segmented endopodite, 2nd endopodial segment not divided and with no male bristles. Seta z_1 transformed into long claw almost equalling (0.94) to length of claw G_2, setae z_2 and z_3 very short. Claw G_1 less than half length of claws G_2 and z_1. Claw G_m approximately 3/4 length of claw G_M. Claw G_2 about 1.6 and claw G_M about 1.3 length of the anterior margin of 2nd undivided endopodial segment.

Mandible, Md (Fig. S3B, Fig. S4C) consists of the coxal plate and a 4-segmented palp Mdp. Coxa typically elongated and heavily chitinized with the masticatory part bearing one seta, seven strong teeth interspaced with sets of diverse setae and one outer lateral strong claw. First Mdp podomere externally with the exopodite plate. Inner margin of 2nd podomere of Mdp with three setae of the so-called setal group, beta seta and one neighbouring seta. Outer edge of this segment with two apical setae. Externo-distal gamma-seta on 3rd podomere smooth. Anterior claw of the terminal podomere amounting to 2.3 and posterior to 1.9 of the L of the 3rd palp podomere. The relative L of one distal seta on the 4th palp podomere 1.7 of the L of the 3rd palp podomere.

Maxillula (Fig. S4D) and female fifth limb with morphology typical of the subfamily.

Fifth limb endopodite palps (clasping organs) of male asymmetrical. Right palp (Fig. 2D, Fig. S4E) rounded, widening distally, more inflated than left, bearing two sup-apical setae. Left palp (Fig. 2F) distally narrower than right, with finger-like distal end bearing two sub-apical setae.

Walking leg, L6 (Fig. S3C) five segmented. Setae e, f and g smooth (length ratio $e \approx f > g$). Terminal segment sub-rectangular in shape, with two short (h_1, h_3) setae, and h_2 seta long and claw-like.

Cleaning leg, L7 (Fig. S3D, Fig. S4F) 4-segmented, endopodial segments EII and EIII fused. Protopodite with three setae (d_1, d_2 and d_p). Short terminal seta h_1 twice the terminal segment and only slightly curved. Relative lengths of three apical setae related to endopodial segment EI as: $h_1 = 0.2$, $h_2 = 1.37$, $h_3 = 1.37$. Seta g as 0.77 of the length of EI.

Uropodal ramus, CR (Fig. 2C, Fig. S4F, Fig. S5A) with anterior claw G_a serrated and strongly curved, while posterior G_p remarkably reduced, less than half of G_a. Anterior seta s_a short, posterior seta s_p c. 1.5 the length of s_a.

Female genital lobe protruding and rounded (Fig. S3E, Fig. S5A).

Hemipenis (Fig. 2E, Fig. S5C-D). Outer lobe (a) with sub-rectangular distal end. Medial lobe (h) is the longest and distally roundly pointed. Inner lobe (b) well developed, distally broadly rounded, with a distinct, acuminated expansion oriented to the postero-ventral end of the body. M-process with a small proximal plate, and a weakly sclerotized and elongated distal part.

Zenker organ (Fig. S5B) with seven rosettes of chitinous spines.

Table S1

Data on species used in molecular analyses: their origins, haplotypes and GenBank Accession numbers of the obtained sequences.

**Species**	**Locality, country**	***COI* haplotypes with species codes (number of specimens)**	**GenBank Accession numbers**
Pseudocandona movilaensis sp. nov.	Movile Cave, Romania	PMOV1(3)	MN013132
Pseudocandona albicans	Gdansk, Poland	PALB1(1), PALB3(1)	MN013121, MN013122
	Oslo, Norway	PALB2(3)	MN013123
Pseudocandona compresa	Lake Ptasi Raj-Gdansk, Poland	PCOM1(2), PCOM2(2), PCOM3(2), PCOM4(1)	MN013099, MN013100, MN013101, MN013102
Pseudocandona hartwigi	Lake Otomińskie-Gdansk, Poland	PHAR1(2)	MN013133
Pseudocandona marchica	Oslo, Norway	PMAR1(1), PMAR2(2)	MN013130, MN013131
Typhlocypris eremita	Gilau, Romania	TERE1(1), TERE2(1), TERE3(4), TERE4(1), TERE5(1)	MN013095, MN013096, MN013097, MN013098
Typhlocypris sywulai	Duderina Jama, Croatia	TSYW1(1), TSYW2(1), TSYW3(1)	MN013093, MN013094
Candona candida	Gdańsk, Poland	CCAN1(1)	MN013107
	Łączyński Młyn, Poland	CCAN2(2)	MN013105
	Lake Raduńskie Górne, Poland	CCAN3(2)	MN013106
Candona weltneri	Lake Nierzostwo, Poland	CWEL1(1), CWEL2(1)	MN013103, MN013104

Table S2

Mean interspecific genetic distance calculated with Kimura 2-parameter method between ostracod species analyzed in this study (for species codes see Table S1).

Species	PMOV	PMAR	PHAR	PALB	PCOM	TERE	TSYW	CCAN
PMOV								
PMAR	0.150							
PHAR	0.149	0.161						
PALB	0.193	0.211	0.196					
PCOM	0.207	0.223	0.208	0.218				
TERE	0.227	0.237	0.213	0.262	0.222			
TSYW	0.244	0.205	0.224	0.235	0.220	0.092		
CCAN	0.256	0.252	0.265	0.261	0.261	0.238	0.242	
CWEL	0.240	0.238	0.228	0.221	0.201	0.259	0.256	0.167

Table S3

Genetic distance calculated with Kimura 2-parameter method between haplotypes of the studied ostracod species (for species and haplotype codes see Table S1).

Haplotype	PMA<br>R<br>1	PMA<br>R<br>2	PAL<br>B<br>1	PAL<br>B<br>3	PAL<br>B<br>2	CWE<br>L<br>1	CWE<br>L<br>2	CCA<br>N<br>2	CCA<br>N<br>3	CCA<br>N<br>1	PMO<br>V<br>1	TERE<br>1	TERE<br>2	TERE<br>4	TERE<br>3	PHA<br>R<br>1	PCO<br>M<br>1	PCO<br>M<br>2	PCO<br>M<br>3	PCO<br>M<br>4	TSY<br>W<br>1	TSY<br>W<br>2
PMAR2	0.002																					
PALB1	0.214	0.217																				
PALB3	0.209	0.212	0.009																			
PALB2	0.207	0.209	0.005	0.007																		
CWEL1	0.235	0.238	0.227	0.219	0.222																	
CWEL2	0.238	0.240	0.222	0.219	0.217	0.005																
CCAN2	0.251	0.253	0.260	0.262	0.265	0.168	0.166															
CCAN3	0.248	0.251	0.257	0.264	0.262	0.168	0.161	0.007														
CCAN1	0.254	0.256	0.257	0.260	0.263	0.170	0.168	0.012	0.012													
PMOV1	0.149	0.151	0.197	0.192	0.190	0.237	0.243	0.256	0.254	0.257												
TERE1	0.235	0.238	0.261	0.263	0.258	0.261	0.258	0.239	0.239	0.239	0.225											
TERE2	0.235	0.238	0.261	0.263	0.258	0.258	0.255	0.236	0.236	0.236	0.225	0.011										
TERE4	0.238	0.241	0.264	0.266	0.261	0.261	0.258	0.239	0.234	0.234	0.228	0.009	0.013									
TERE3	0.235	0.238	0.266	0.269	0.264	0.264	0.261	0.241	0.241	0.241	0.230	0.014	0.004	0.016								
PHAR1	0.160	0.163	0.195	0.200	0.193	0.228	0.228	0.267	0.259	0.268	0.149	0.214	0.209	0.217	0.214							
PCOM1	0.226	0.228	0.225	0.220	0.217	0.203	0.205	0.265	0.263	0.265	0.210	0.225	0.230	0.225	0.235	0.212						
PCOM2	0.211	0.213	0.223	0.217	0.215	0.203	0.200	0.257	0.254	0.262	0.205	0.212	0.217	0.212	0.222	0.200	0.037					
PCOM3	0.223	0.226	0.222	0.217	0.215	0.200	0.203	0.262	0.260	0.263	0.207	0.222	0.227	0.222	0.232	0.215	0.002	0.035				
PCOM4	0.226	0.228	0.220	0.214	0.212	0.196	0.198	0.260	0.257	0.260	0.205	0.214	0.220	0.214	0.225	0.207	0.007	0.029	0.005			
TSYW1	0.206	0.208	0.235	0.237	0.232	0.259	0.254	0.247	0.242	0.244	0.247	0.098	0.096	0.098	0.092	0.225	0.227	0.214	0.224	0.222		
TSYW2	0.203	0.206	0.240	0.242	0.237	0.259	0.254	0.244	0.239	0.241	0.244	0.094	0.092	0.094	0.087	0.225	0.224	0.212	0.222	0.219	0.004	
TSYW3	0.201	0.204	0.230	0.233	0.228	0.257	0.251	0.244	0.239	0.239	0.242	0.092	0.090	0.092	0.086	0.221	0.223	0.210	0.220	0.217	0.005	0.009

Figure S1

Pseudocandona movilaensis sp. nov. A. External view of female left valve. B. External view of female right valve. C. External view of male left valve.

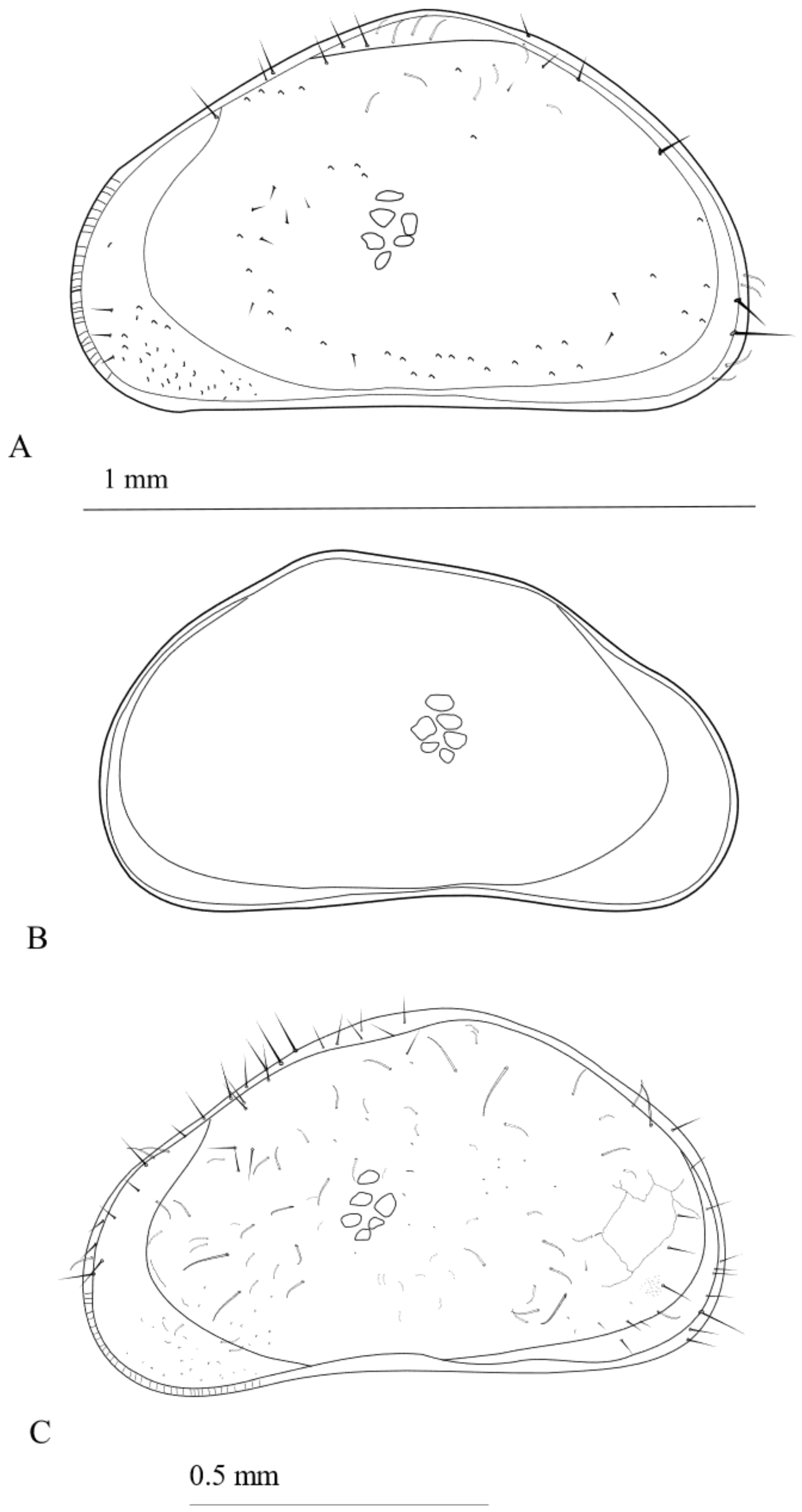

Figure S2

Pseudocandona movilaensis sp. nov. juvenile carapace and valves. A. Dorsal view of juvenile A-1, anterior to left. B. External view of juvenile A-1 left valve. C. Internal view of juvenile A-1 right valve. D. External view of juvenile A-1 right valve. E. Internal view of juvenile A-2 left valve. F. External view of juvenile A-2 left valve. G. internal view of juvenile A-2 right valve. H. External view of juvenile A-2 right valve. I. internal view of juvenile A-3 left valve. J. External view of juvenile A-3 right valve. K. Internal view of juvenile A-4 left valve. L. Internal view of juvenile A-4 right valve.

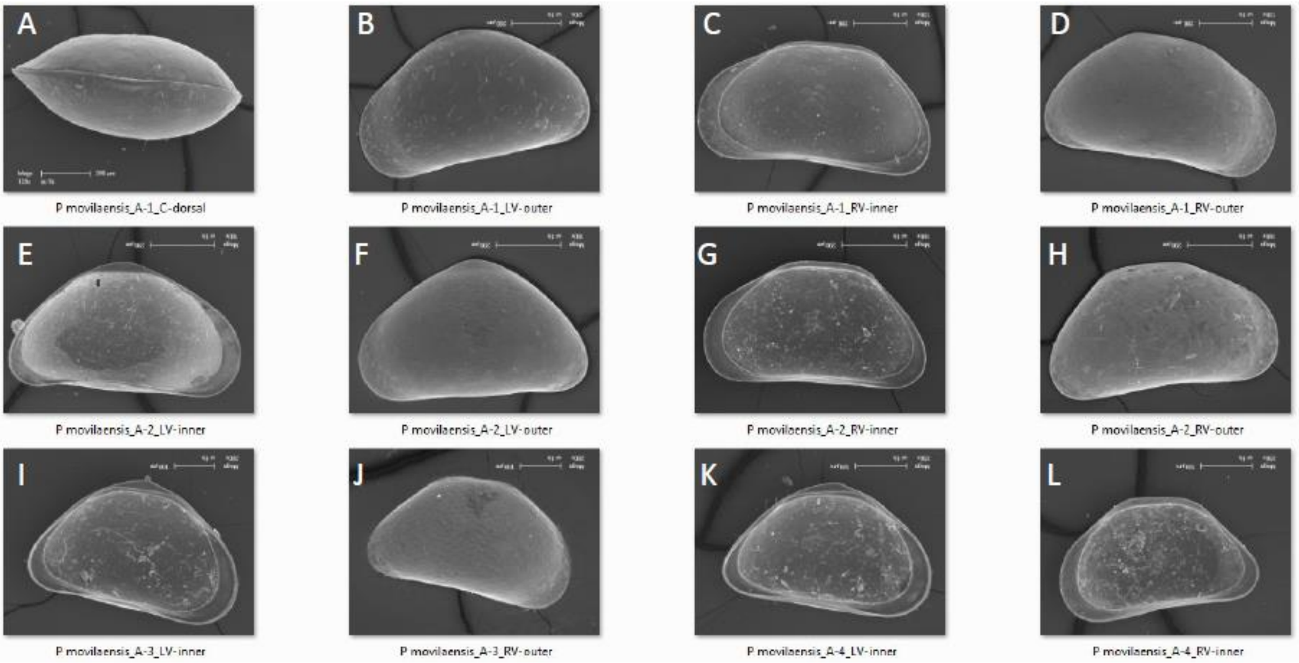

Figure S3

Pseudocandona movilaensis sp. nov. (female) A. Antennule. B. Mandibula. C. Sixth limb (walking leg). D. Seventh limb (cleaning leg). Scale bars (100 μm).

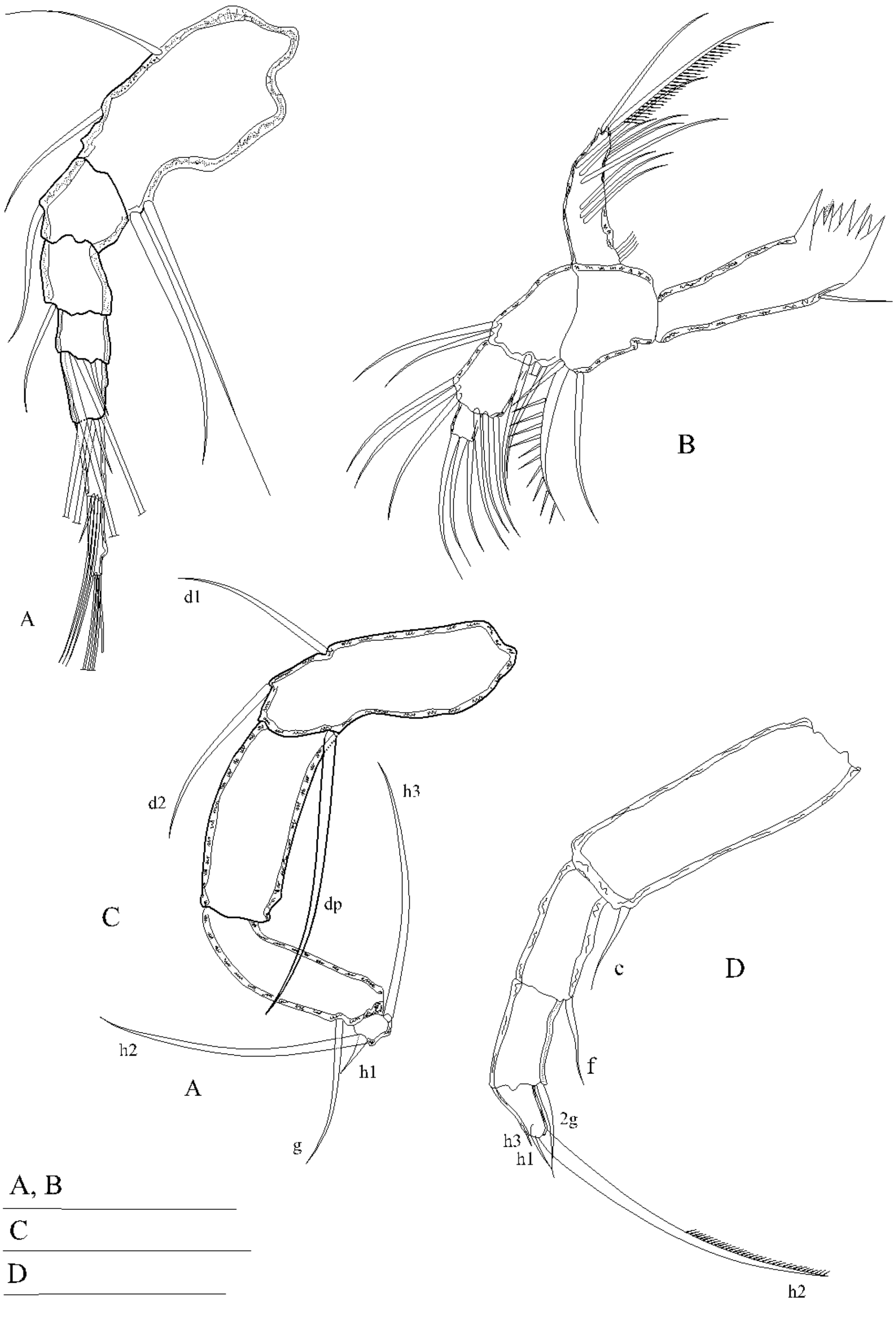

Figure S4

Pseudocandona movilaensis sp. nov. (male). A. Antenna. B. Mandibula. C. Maxilla. D. Right fifth limb. E. Seventh limb (cleaning leg). F. Uropodal rami.

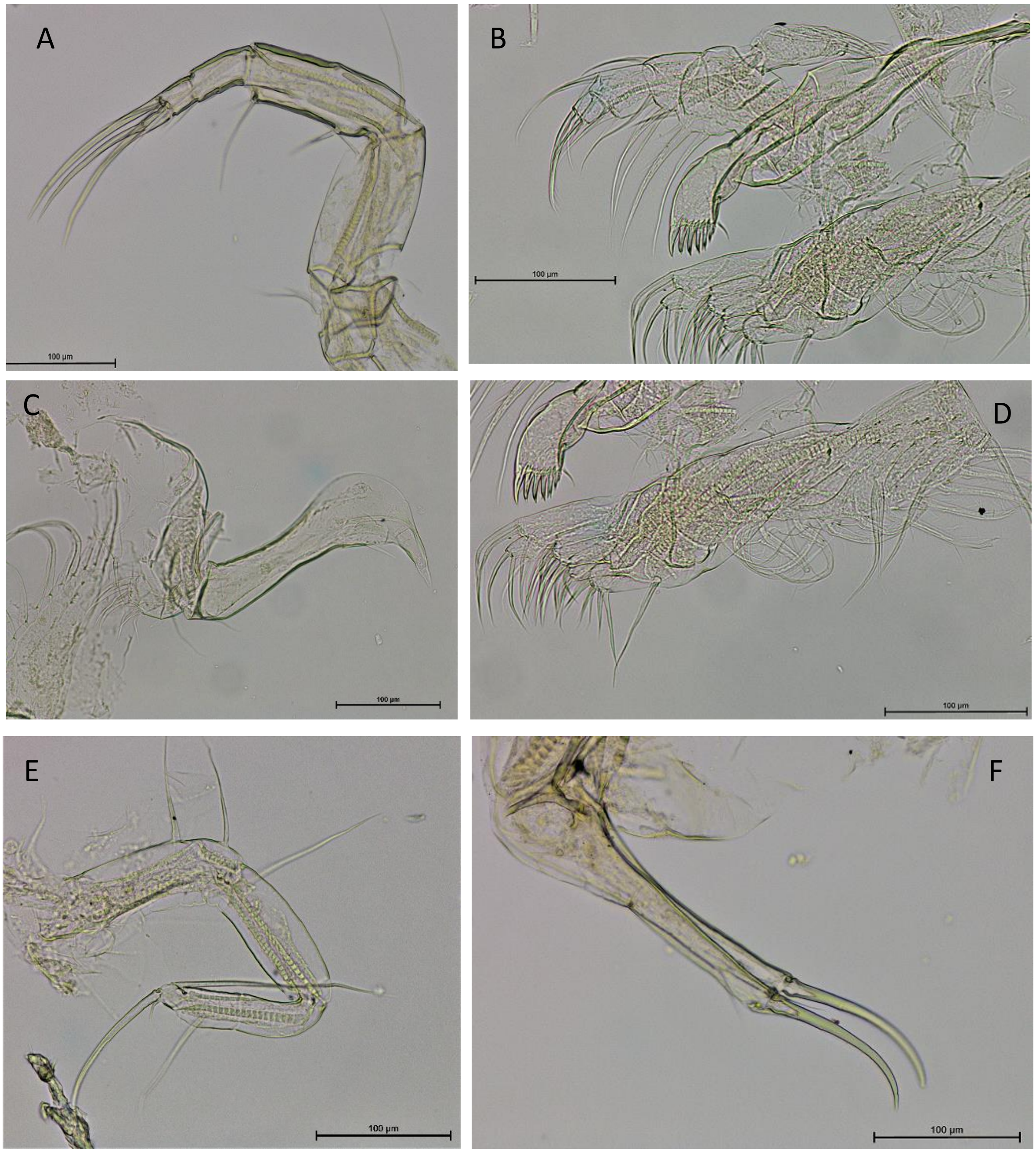

Figure S5

Pseudocandona movilaensis sp. nov. A. Female uropodal rami and genital lobes. B. Male Zenker organ and uropodal rami. C. Hemipenis. D. Detail of the distal part of the ductus ejaculatorius in the erect position.

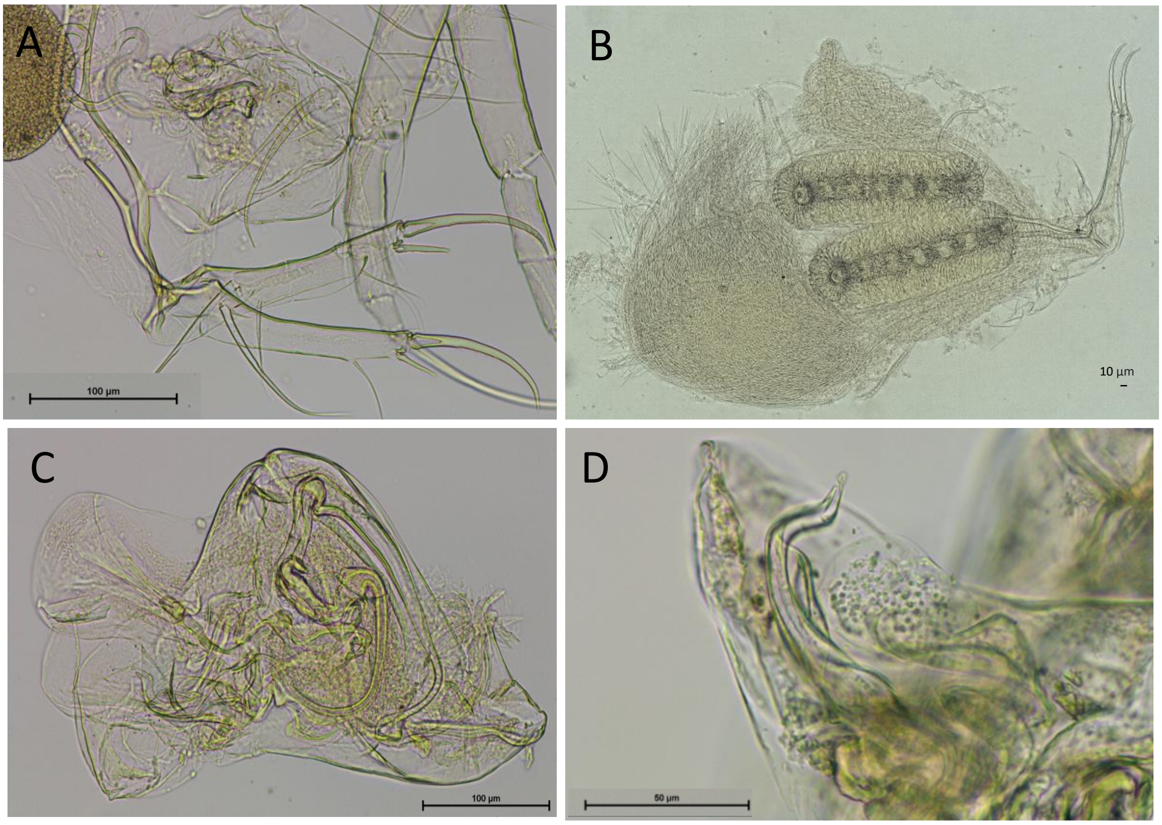

Supplement: Supplementary file 1 — Supplementary Information. [file 41598_2023_32573_MOESM1_ESM.pdf]
